# Very Slowly Progressive Microscopic Polyangiitis: Comparative Analysis with Rapidly Progressive Forms

**DOI:** 10.7759/cureus.62282

**Published:** 2024-06-13

**Authors:** Narumichi Iwamura, Kanako Tsutsumi, Yuki Ueno, Yasuhisa Tamura, Toshiaki Nakano

**Affiliations:** 1 Department of Nephrology, Japan Community Health Care Organization Kyushu Hospital, Kitakyushu, JPN; 2 Department of Nephrology, Steel Memorial Yahata Hospital, Kitakyushu, JPN; 3 Department of Medicine and Clinical Science, Graduate School of Medical Sciences, Kyushu University, Fukuoka, JPN

**Keywords:** chronic inflammation, slowly progressive mpa, rapidly progressive glomerulonephritis (rpgn), myeloperoxidase-anti-neutrophil cytoplasmic antibodies (mpo-anca), anca-associated vasculitis (aav), microscopic polyangiitis (mpa)

## Abstract

Microscopic polyangiitis (MPA) is predominantly characterized by rapidly progressive glomerulonephritis (RPGN) associated with myeloperoxidase anti-neutrophil cytoplasmic antibodies (MPO-ANCA). Nonetheless, up to 30% of cases of ANCA-associated vasculitis (AAV) may exhibit a more indolent progression toward renal failure, an aspect less frequently discussed and understood in medical literature. This study seeks to clarify the clinical and pathological distinctions between the slowly and rapidly progressive forms of MPA, thereby enhancing understanding of their distinct pathogeneses and treatment responses. We conducted a comparative analysis of two patients diagnosed with MPA under the 2022 American College of Rheumatology/the European Alliance of Associations for Rheumatology (ACR/EULAR) classification. Evaluations included laboratory tests such as serum creatinine levels, serology for MPO-ANCA, and renal biopsies. Patient 1 exhibited a mere 1.07% decrease in estimated glomerular filtration rate (eGFR) over 6 months, significantly below the RPGN threshold, and demonstrated sclerotic glomerular pathology without active inflammation. This patient also showed lower levels of MPO-ANCA, Birmingham Vasculitis Activity Score (BVAS), and C-reactive protein. Conversely, Patient 2 experienced an 89.9% reduction in eGFR over the same timeframe, accompanied by acute systemic inflammation. The comparative clinical analysis of these cases illuminates clear differences in disease activity. Slowly progressive MPA is marked by lesser disease activity that fosters chronic inflammation, leading to a more gradual decline in renal function. Early diagnosis, facilitated by initial measurements of MPO-ANCA, can enhance disease management and improve patient outcomes.

## Introduction

Davies et al. first described the relationship between anti-neutrophil cytoplasmic antibodies (ANCA) and the etiology of necrotizing and crescentic glomerulonephritis [1]. In 1998, Falk and Jennette postulated that myeloperoxidase (MPO)-ANCA is a type of pauci-immune crescentic glomerulonephritis and a possible etiology of systemic vasculitis [2]. Microscopic polyangiitis (MPA) is defined as necrotizing vasculitis, mainly small vessel vasculitis without granulomatous lesions. Most MPA cases are positive for MPO-ANCA, which is important in pathogenesis. Renal involvement is usually associated with rapidly progressive glomerulonephritis (RPGN) [3]. This renal syndrome is characterized by a rapid decline in glomerular filtration rate, activity of urine sediment in the presence of erythrocyte casts, and histological findings of non-immune crescentic glomerulonephritis [4]. RPGN requires early induction treatment with glucocorticoids and immunosuppressive drugs, such as cyclophosphamide or rituximab, to stop vasculitis activity and restore renal function [5]. Nevertheless, up to 30% of patients with ANCA-associated vasculitis (AAV) with renal involvement manifest a slow course, with less severe symptoms than those of RPGN, and a slowly progressive toward end-stage renal disease [4]. This slowly progressive form of MPA has rarely been discussed in the literature [6], with only an observational study [7] and anecdotal cases [8,9]. Trivioli et al. conducted a cross-sectional observational study including 41 patients of slow progressive AAV [7]. They selected patients with two or more renal function assessments performed within 6 months before diagnosis and analyzed their GFR deterioration. They defined slow progression as eGFR reduction > 25% but ≦50% over the 6-month period preceding diagnosis. Over a 6-month period, we observed an eGFR decline rate of 1.07% (with eGFR measuring 29.3 ml/min/1.73m² 23 weeks prior to diagnosis and 29.0 ml/min/1.73m² at the time of diagnosis), which was an even slower rate of eGFR decline than previously reported in cases of slowly progressive AAV. The case was consistent with MPA both clinically and pathologically and did not contradict the clinical characteristics of previously reported cases of slowly progressive AAV otherwise the rate of renal decline, leading us to consider it as a case of very slowly progressive MPA. This unusual phenotype probably accounts for a small proportion of AAV and may be difficult to recognize because it is not typical of vasculitis and also occurs in other more prevalent renal diseases, such as chronic glomerulonephritis and hypertensive nephrosclerosis. We report a case of slowly progressive MPA with sclerotic renal pathology and another case of rapidly progressive MPA with similar renal pathology. We report a case of very slowly progressive MPA with sclerotic renal pathology, compared with another case of rapidly progressive MPA with similar renal pathology.

## Case presentation

Case 1: Slowly progressive microscopic polyangiitis (MPA)

**Figure 1:**
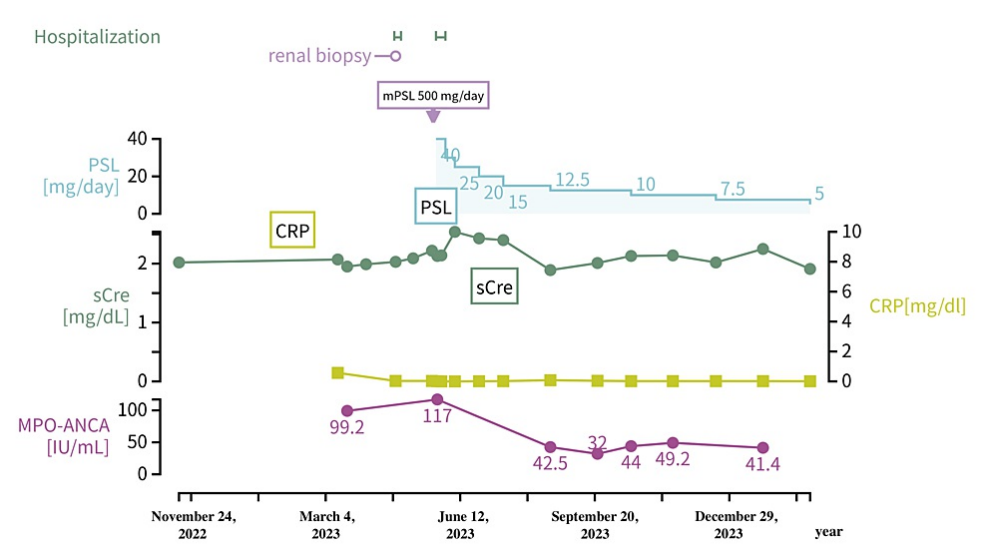
Time course of Case 1 mPSL: methylprednisolone; PSL: prednisolone; sCre: serum creatinine; CRP: C-reactive protein; MPO-ANCA: myeloperoxidase anti-neutrophil cytoplasmic antibodies

The patient was a 51-year-old man with no comorbidities or pre-existing medical conditions other than gastric ulcer and cataract disease. He had no renal function abnormalities detected during school health checkups. His last workplace physical examination was 10 years ago, at which time no abnormalities in renal function were noted. His home blood pressure has been consistently around 130/80 mmHg. On November 15, 2022, a preoperative examination for cataract surgery revealed renal dysfunction with serum creatinine 2.02 and eGFR 29.3 ml/min/1.73 m^2^, but no urinalysis was performed. No symptomatic infections or vaccinations had occurred within 6 months of these tests. The changes in Patient 1’s renal function and course of treatment are shown in Figure 1.

He was referred to a nearby clinic on March 13, 2023, for the follow-up assessment of renal function. Blood tests showed a serum creatinine level of 2.07 mg/dl, and he was referred and admitted into our hospital on April 24, 2023 (Day 0) for close examination and treatment of renal dysfunction.

On physical examination, no arthralgia or myalgia, purpura, subcutaneous hemorrhage, edema, or obvious neurological abnormalities were observed. The results of laboratory tests on admission are presented in Table 1.

**Table 1:** Laboratory data of Cases 1 and 2 eGFR: estimated glomerular filtration rate; anti-GBM antibody, anti-glomerular basement membrane antibody; PR3-ANCA, proteinase 3-anti-neutrophil cytoplasmic antibody cytoplasmic antibody; MPO-ANCA: myeloperoxidase-anti-neutrophil

	Case 1 (May 22, 2023)	Case 2 (August 25, 2023)	Reference Range
Blood cell counting			
Hemoglobin	11.5	7.90	13.7-16.8 g/dl
Platelet	201	181	158-348 k/μl
White blood cell	6.30	10.8	3.30-8.60 k/μl
Neutrophil	64	72	38-59%
Chemistry			
Total protein	6.4	6.5	6.6-8.1 g/dl
Albumin	3.9	2.7	4.1-5.1 g/dl
Creatinine kinase	88	12	59-248 U/l
Aspartate aminotransferase	13	15	13-30 U/l
Alanine aminotransferase	9	7	10-42 U/l
Lactate dehydrogenase	170	185	124-222 U/l
Alkaline phosphatase	59	74	38-113 U/l
γ-glutamyl transpeptidase	18	34	13-64 U/l
Creatinine	2.22	1.95	0.650-1.07 mg/dl
eGFR	26	20	> 60 mL/min/1.73m^2^
Uric acid	8.3	5.8	3.7-7.8 mg/dl
Urea nitrogen	34	24	8.0 -20 mg/dl
Sodium	141	136	138-145 mEq/l
Potassium	4.4	3.2	3.6-4.8 mEq/l
Chlorine	109	97	101-108 mEq/l
Total-bilirubin	0.70	0.70	0.400-1.50 mg/dl
Immunology			
C-reactive protein	0.030	5.0	0.00-0.14 mg/dl
Complement component 3	84	126	73-138 mg/dl
Complement component 4	18	26	11-31 mg/dl
50% hemolytic unit of complement	25	N/A	32-58 U/ml
Antinuclear antibody	Negative	negative	< 40 l
Rheumatoid factor	Negative	144	< 15 IU/ml
Immunoglobulin G	933	1686	870-1700 mg/dl
Immunoglobulin A	134	N/A	110-410 mg/dl
Immunoglobulin M	71	119	33-260 mg/dl
PR3-ANCA	< 1.0	< 1.0	< 2.0 IU/ml
MPO-ANCA	99.2	276	< 3.5 IU/ml
Anti-GBM antibody	< 2.0	< 2.0	< 7.0 IU/ml
Urinalysis			
Urinary protein/urinary creatinine	0.44	0.99	< 0.05 g/gCre
Urinary blood	10-19	1-4	0-4/high-power field
Hyaline cast	50-99	0	< 1/high-power field
Epithelial cast	10-19	0	< 1/high-power field
Granular cast	20-29	0	< 1/high-power field
Waxy cast	5	0	< 1/high-power field
Dysmorphic red blood cell	±	-	< 1/high-power field

**Figure 2:**
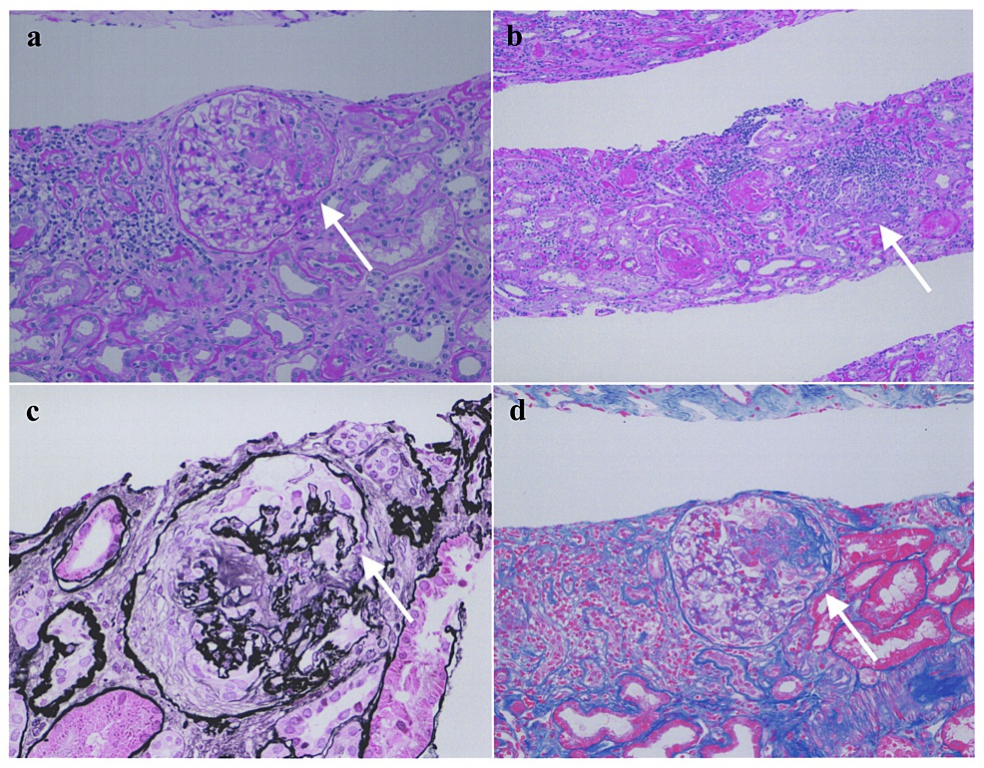
Kidney biopsy of Case 1 (a) One glomerulus reveals segmental sclerosis, with segmental endocapillary hypercellularity + cellular crescent. Periodic acid-Schiff stain, ×200 magnification; (b) Tubulointerstitium is severely involved with fibrosis and tubular atrophy in about 50% of the cortex. Periodic acid-Schiff stain, ×100 magnification; (c) Focal double contours of the glomerular basement membranes are seen. Periodic acid-methenamine-silver stain, × 400 magnification; (d) One glomerulus reveals segmental sclerosis, with segmental endocapillary hypercellularity plus cellular crescent. Masson trichrome stain, ×200 magnification.

The patient’s blood tests on admission revealed a serum creatinine level of 2.22 mg/dl and a high MPO-ANCA of 99.2 IU/ml. Urinalysis showed a urinary protein/urinary creatinine rate of 0.44 g/gCre, and urinary sediment indicated occult blood at 10-19 high-power field (HPF), epithelial columns at 10-19/HPF, granular columns at 20-29/HPF, and waxy columns at 5/HPF. Both fecal occult blood tests were negative, and computed tomography revealed no obvious hemorrhagic or interstitial lesions in the lung fields. An ultrasound scan revealed a right kidney measuring 95 × 47 mm and a left kidney measuring 94 × 49 mm, with no obvious renal morphological abnormalities. Pathological images from the renal biopsy performed on April 25, 2023, are shown in Figure 2.

Of the 29 glomeruli specimens, 21 had total nodular sclerosis. One showed partial endothelial cell proliferation and cellular crescents, whereas three showed fibrous crescents. The tubular interstitial was highly fibrotic, with tubular atrophy in 50% of the renal cortex and mild lymphocytic infiltration in 10% of the renal cortex. Fluorescent antibody analysis revealed no specific immune complex depositions. Electron microscopy also showed no electron-dense deposit. Based on the above findings, especially sclerotic glomeruli with sclerotic and fibrotic interstitial and fibrous crescents, the patient had a relatively long course of ANCA-associated nephritis with positive MPO-ANCA.

The patient did not present with RPGN, as his estimated glomerular filtration rate (eGFR) decreased only 11.2%, from 29.3 ml/min/1.73 m^2^ to 26 ml/min/1.73 m^2^, in 6 months since the initial diagnosis. Therefore, this case did not meet the 1998 Japanese Ministry of Health, Labour and Welfare (MHLW) criteria for MPA [10], which lists RPGN as one of the major syndromes. This case meets the diagnostic criteria for primary systemic vasculitis, showing symptoms consistent with AAV, histologically confirmed vasculitis, positive MPO-ANCA, and exclusion of other diseases. According to Watts’ classification algorithm, it exhibits clinical signs and histological findings consistent with small vessel vasculitis. With no substitute markers for granulomatosis with polyangiitis (GPA) observed, the case was categorized as MPA [11].

This case was also classified as MPA according to the 2022 ACR/EULAR classification for MPA, which is based on the presence of positive MPO-ANCA and pauci-immune type glomerulonephritis [12]. His symptoms and laboratory findings were inconsistent with polyarteritis nodosa, GPA, EGPA; collagen diseases, such as systemic lupus erythematosus (SLE) and rheumatoid arthritis (RA); and IgA vasculitis. We diagnosed the case as slowly progressive MPA based on the ruling out of other plausible differential diseases, an elevated MPO-ANCA, and renal pathology supporting ANCA-associated nephritis. The Birmingham Vasculitis Activity Score (BVAS) version 3 [13] before the treatment initiation was 13/63. Using the criteria proposed by Berden et al. [14], the disease was further classified as the sclerosing type, which has the poorest prognosis. As recommended in the Japan Evidence-Based Rapidly Progressive Nephritis Syndrome (RPGN) Practice Guidelines 2020 [15], the patient was treated with corticosteroids to induce remission.

At 29 weeks after the initial examination (Day 27), blood tests showed a serum creatinine level of 2.22 mg/dl; eGFR of 26 ml/min/1.73 m^2^; MPO-ANCA at 117 U/ml; urine protein/urine creatinine of 0.81 g/gCre; urinary erythrocytes at 10-19 /HPF; granule columns at 10-19/HPF; two erythrocyte columns; and presence of deformed erythrocytes. On the same day, he received methylprednisolone (mPSL) 500 mg/day for 3 days, followed by PSL 40 mg/day (0.68 mg/kg/day) for 7 days for ANCA-associated nephritis. On Day 37, the patient was discharged after a dose reduction for PSL to 30 mg/day and was followed up at the outpatient clinic thereafter. The PSL dose was reduced every 2-4 weeks at a rate of 2.5 to 5 mg/day. At 4 months after the initiation of treatment (Day 150), the oral steroid dose was 7.5 mg. Blood tests at this point showed a clear decrease in MPO-ANCA at 32.0 U/ml. Urinalysis showed a urine protein/creatinine of 0.33 g/gCre, urinary sediment of 1-4 erythrocytes/HPF, and disappearance of columns, indicating significant improvement in urinary findings. The BVAS at this point was 5 points, indicating a meaningful reduction in disease activity, although not a complete remission. However, improvement in renal function was limited, with a serum creatinine level of 2.01 mg/dl and eGFR of 29 ml/min/1.73 m^2^.

Case 2: Rapidly progressive MPA

**Figure 3:**
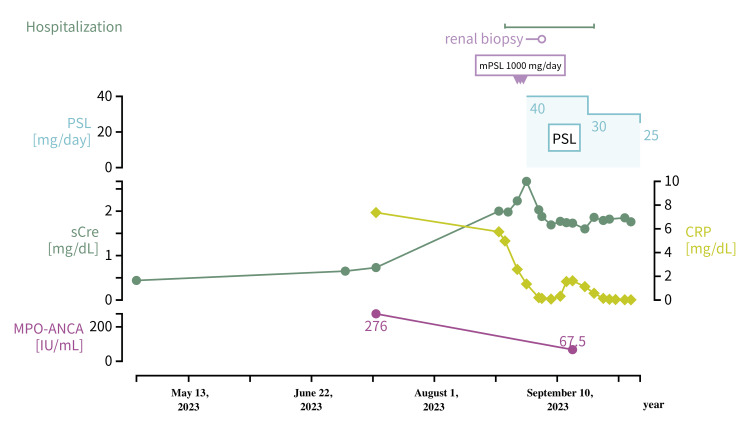
Time course of Case 2 mPSL: methylprednisolone; PSL: prednisolone; sCre: serum creatinine; CRP: C-reactive protein; MPO-ANCA: myeloperoxidase anti-neutrophil cytoplasmic antibodies

The patient was a 69-year-old woman with type 2 diabetes and hypertension. She had no family history of renal failure and had not been diagnosed with any abnormalities at medical checkups at work. A routine follow-up blood test on April 26, 2023, revealed a serum creatinine level of 0.44 mg/dl and eGFR of 104.9 ml/min/1.73 m^2^. Urinalysis revealed urine occult blood ± and urine protein negative. The patient had fatigue and anorexia, edema in both lower legs, and a weight loss of 5 kg in 2 months. She had no history of symptomatic infection or vaccination in the 6 months prior. The changes in Patient 2’s renal function and the course of treatment are shown in Figure 3.

She was referred to our hospital on July 13, 2023, for close examination and treatment for worsening leg edema and weight loss. At the initial visit, blood tests showed a serum creatinine level of 0.65 mg/dl and eGFR 68 ml/min/1.73 m^2^. Urinalysis did not reveal any urine occult blood or proteins. The eGFR had decreased from 104.9 ml/min/1.73 m^2^ to 15 ml/min/1.73 m^2^ in 4 months since the last examination before the onset of symptoms, and the rate of decrease from the baseline eGFR was 89.9%, which was thought to be rapidly progressive. The blood test performed on July 13, 2023, showed a slow decline in renal function with a serum creatinine level of 0.73 mg/dl and an elevated inflammatory response with a C-reactive protein (CRP) of 7.37 mg/dl. Antinuclear antibodies and PR3-ANCA were negative, but MPO-ANCA was elevated at 276 IU/ml. The patient was admitted to our hospital on August 22, 2023 (Day 0), with suspected ANCA-related vasculitis.

On admission, a physical examination revealed severe anemia of the eyelid conjunctiva. Purpura on the left lower leg and severe indurated edema on both lower legs were noted. Neurological examination revealed normal tactile sensation but a decreased warmth and pain sensation of 5/10 in the left lateral plantar nerve and peroneal nerve areas. Manual muscle strength testing revealed differences between the left and right extensor hallucis longus, flexor digitorum longus, and tibialis posterior muscles, with muscle weakness ranging from 2 to 3 out of 5 points, indicating mononeuritis multiplex. Patient 2’s on-admission laboratory data are presented in Table 1. Her blood tests showed decreased renal function with a serum creatinine level of 1.95 mg/dl; eGFR of 20 ml/min/1.73 m^2^ elevated CRP level of 4.98 mg/dl; MPO-ANCA of 276 IU/ml. Anemia was noted with hemoglobin level of 6.0 g/dl. Urinalysis revealed proteinuria of ±, erythrocytes in the urine at 1-4/HPF, and no significant columns in the urine sediment. Computed tomography of the lung field showed reticular shadows under the pleura at the lung bases, indicating interstitial pneumonia. Upper and lower gastrointestinal endoscopies revealed no significant abnormalities.

**Figure 4:**
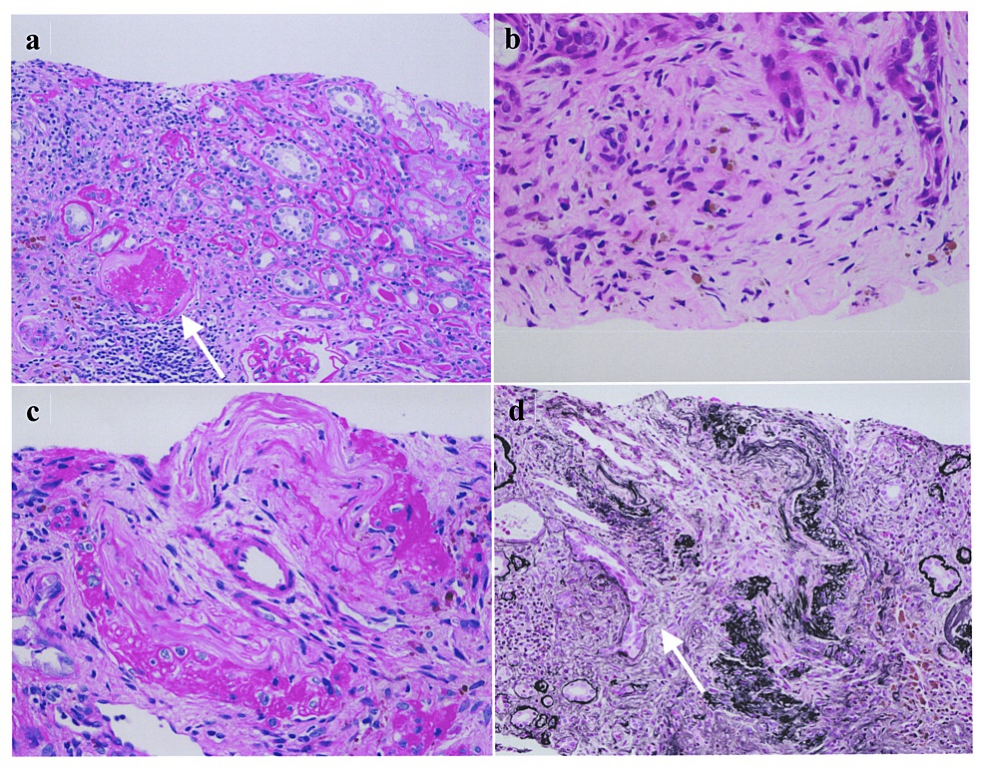
Kidney biopsy of Case 2 (a) One glomerulus is collapsed with infiltration of lymphocytes. Hematoxylin and eosin stain, ×200 magnification; (b) Lymphocyte and eosinophil infiltration are noticed in about 20% of the cortex. Hematoxylin and eosin stain, ×400 magnification; (c) Arterioles are mildly sclerotic with segmental hyaline change in about 10% of the arterioles. Hematoxylin and eosin stain, ×400 magnification; (d) One interlobular artery shows edema and fibrosis of intima with lymphocyte and eosinophil infiltration and rupture of the arterial wall with hemosiderin deposition. Periodic acid-methenamine-silver stain, ×200 magnification.

A skin biopsy of purpura in the left lower leg on Day 7 showed mild infiltration of inflammatory cells around the small blood vessels, but no neutrophil or eosinophil infiltration and no evidence of vasculitis. A bronchopulmonary biopsy performed on Day 8 showed mild infiltration of chronic inflammatory cells around small blood vessels, but none in the submucosa or other specific inflammatory findings. A renal biopsy performed on Day 14 showed total nodular sclerosis in 16 of 32 glomeruli observed. Two glomeruli had mild mesangial cell proliferation, one of which had mild endothelial cell proliferation and the other had collapsed. No proliferative lesions were observed in other glomeruli (Figure 4).

Tubulitis and peritubular capillaritis were also found; one interlobular artery showed intimal edema and fibrosis with lymphocyte and eosinophil infiltration, and destruction of the arterial wall with hemosiderin deposition. Immunofluorescence analysis revealed no specific deposits on the glomerular wall or mesangial lesions.

This case meets the 1998 Japanese Ministry of Health, Labour and Welfare MHLW criteria for MPA, showing RPGN, interstitial pneumonia, purpura, and polyneuritis. This case meets the diagnostic criteria for primary systemic vasculitis, showing symptoms consistent with AAV, histologically confirmed vasculitis, positive MPO-ANCA, and exclusion of other diseases. According to Watts’ classification algorithm, it exhibits clinical signs and histological findings consistent with small vessel vasculitis. With no substitute markers for GPA observed, the case was categorized as MPA [11].

This case was also classified as MPA according to the 2022 ACR/EULAR classification for MPA, which is based on the presence of positive MPO-ANCA, pauci-immune type glomerulonephritis, and fibrosis on chest image [12]. The patient’s symptoms and laboratory findings were consistent with those of polyarteritis nodosa, GPA, eosinophilic GPA, systemic lupus erythematosus, RA, and IgA vasculitis. The decline rate of eGFR of this patient in a 6-month period was 89.9%, from 104.9 ml/min/1.73 m^2^ to 15.0 ml/min/1.73 m^2^, thereby exhibiting RPGN. Because of the high MPO-ANCA level of 276 IU/ml and the negative evidence of other diseases causing renal dysfunction, we diagnosed the case as MPA. The BVAS version 3 before starting treatment was 27/63 [13], which indicated high disease activity. Histological findings of renal pathology were also classified as sclerosing [14]. On Day 6 (18 weeks after the initial examination), the patient received mPSL 1000 mg/day for 3 days, followed by PSL 40 mg/day (1 mg/kg/day) for 3 weeks. Blood tests on Day 24 showed a serum creatinine level of 1.73 mg/dl; eGFR of 23 ml/min/1.73 m^2^; and MPO-ANCA of 67.5 IU/ml. Urinalysis revealed a urine protein/creatinine of 0.53 g/gCre; urine sediment < 1 erythrocyte/HPF, and no significant columns. BVAS version 3 was 7/63, indicating a good response to immunosuppressive therapy.

## Discussion

Slowly progressive MPA is rarely discussed in the literature; only a few anecdotal reports have been published to date, all from Japan [8,9]. Trivioli et al. conducted a retrospective observational study of 856 patients with ANCA-associated nephritis, including 383 patients with MPA, to determine the proportion of slowly progressive forms and their clinical and pathological characteristics in AAV [7]. They found that, of 856 patients, 41 (5%) had slowly progressive renal AAV, and all had MPA. Forty-one of the 383 patients with MPA (10.7%) had slowly progressive renal AAV. Although there is no universal definition has yet been established for slowly progressive, we simply defined MPA as an eGFR decline of less than 50% within 6 months as slowly progressive. The 6-month reduction in eGFR in Case 1 and Case 2 was 11.3% and 89.9 %, respectively. In accordance with the aforementioned definitions, Case 1 was considered a slowly progressive MPA, whereas Case 2 was a rapidly progressive MPA. We compared the clinical features of Case 1 with 41 patients of slowly progressive AAV reported by Trivioli et al. [7] (Table 2). According to this study, 61% (25/41) of patients had the renal-limited type of slowly progressive MPA, and the median BVAS that indicated the clinical activity of AAV was 13. Case 1 exhibited renally localized MPA with no pulmonary complications and a BVAS score of 13, which are generally consistent with the characteristics reported by Trivioli et al. They also reported a median serum creatinine level and eGFR at diagnosis of 2.3 mg/dl and 23 ml/min/1.73 m^2^, respectively [7]. In Case 1, serum creatinine and eGFR at diagnosis were 2.22 mg/dl and 23 ml/min/1.73 m^2^, respectively, which are consistent with those in previous reports. Trivioli et al. also found that the dominant pathological type of slowly progressive MPA was sclerotic (12/28) based on the Berden et al. criteria [7]. The pathology of Case 1 was similar to that of sclerotic AAV, 21 of 29 glomeruli in the pathology specimen showed global sclerosis. It confirms the incidence of an MPA subtype with very similar clinical and pathological features in our hospital.

Trivioli et al. conducted a cross-sectional observational study including 41 patients with slow progressive AAV [7]. They selected patients who had undergone two or more renal function assessments within 6 months before diagnosis and analyzed their GFR deterioration. They defined slow progression as an eGFR reduction greater than 25% but less than or equal to 50% over the 6 months preceding the diagnosis. They noted that these limits allowed for differentiation between slow renal course and RPGN, usually defined as an eGFR reduction greater than 50% in up to 3 months, and from kidney impairment with no significant progression [7]. In Case 1, over a 6-month period, we observed an eGFR decline rate of 1.07% (eGFR was 29.3 ml/min/1.73 m² 23 weeks prior to diagnosis and 29.0 ml/min/1.73 m² at the time of diagnosis), indicating an even slower rate of eGFR decline than previously reported. The case was consistent with MPA both clinically and pathologically and did not contradict the clinical characteristics of previously reported cases of slowly progressive AAV otherwise the rate of renal decline, leading us to consider it as a case of very slowly progressive MPA. As this case demonstrates, with an eGFR decline rate over 6 months of less than 25%, indicating a very gradual decline in renal function, it may be necessary to carefully redefine slowly progressive MPA, considering the potential existence of similar cases.

**Figure 5:**
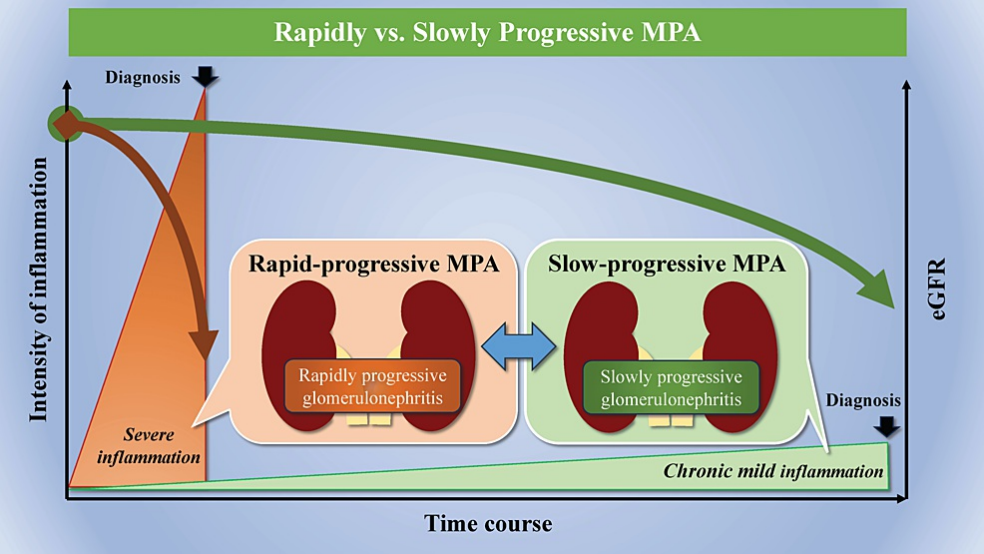
Slowly versus rapidly progressive MPA MPA: microscopic polyangiitis; eGFR: estimated glomerular filtration rate. Image credits: Iwamura N & Tsutsumi K

What is the difference between slowly progressive MPA in Case 1 and rapidly progressive MPA in Case 2? Trivioli et al. considered slowly progressive MPA to have a different pathogenesis than rapidly progressive MPA. They argue that slowly progressive MPA may represent a subset of the disease in which MPO-ANCA promotes renal sclerosis rather than vasculitis, resulting in mild manifestations, slow progression, and accumulation of chronic damage. However, they do not address the reason why most MPO-ANCA-positive patients develop RPGN and a small proportion has slowly progressive renal disease [7]. In contrast, we propose another hypothesis: among the spectrum of MPA cases with various activities, rapidly progressive MPA typifies those cases with high activity, resulting in rapid renal dysfunction due to acute inflammation, whereas slowly progressive MPA is characterized by relatively milder activity that exhibits slower renal dysfunction caused by chronic inflammation (Figure 5).

The term “slowly progressive MPA” is suitable for describing a group of patients exhibiting a slow decline in renal function due to chronic inflammation because of relatively mild MPA activity. This hypothesis is supported by the following reasons. We compared the clinical features of Case 1 with slowly progressive renal decline and Case 2 with rapidly progressive renal decline. Case 1 had renal MPA, whereas Case 2 had skin, nerve, and lung lesions, in addition to renal lesions. Case 1 had a pre-treatment MPO-ANCA of 99.2 U/ml, BVAS of 13, and CRP of 0.030 mg/dl. Conversely, Case 2 had a pre-treatment MPA of 276 U/ml, BVAS of 27, and CRP of 4.98 mg/dl. These facts indicated that the MPA in Case 1 was less active than that in Case 2. Therefore, slowly progressive MPA may be less active than rapidly progressive MPA. Renal pathology revealed that 74% (21/29) of the glomeruli in Case 1 had total nodular necrosis, whereas only 50% (16/32) of the glomeruli in Case 2 had it. While both cases were classified as sclerosing according to the criteria of Berden et al., the proportion of sclerotic glomeruli in Case 1 was clearly higher than that in Case 2, suggesting a longer course of ANCA-associated nephritis.

The optimal management and outcomes of slowly progressive MPA remain unclear. In an observational study by Trivioli et al., 56% (23/41) of patients with slowly progressive ANCA-related nephritis received conventional immunosuppressive therapy including glucocorticoids. In the report studying the outcome of slowly progressive ANCA-associated nephritis [7], 37% (15/41) already had an eGFR under 15 ml/min/1.73 m^2^ at diagnosis, while 15% (6/41) required renal replacement therapy. In a study investigating the outcomes of patients initiated on immunosuppressive therapy, only 59% (20/41) showed an improvement in eGFR ≤ 25% from baseline at the last follow-up. In Case 1, treatment with mPSL 500 mg for 3 days followed by PSL 40 mg/day (0.68 mg/kg/day) resulted in an eGFR improvement of only 11.5% (from 26 to 29 ml/min/1.73 m^2^) from baseline at 4 months after treatment initiation. Conversely, in Case 2, mPSL 1000 mg for 3 days followed by PSL 40 mg/day (1 mg/kg/day) improved eGFR by 53.3% (from 15 to 23 ml/min/1.73 m^2^) from baseline at 5 weeks after treatment initiation. In patients with impaired renal function in slowly progressive MPA, most renal dysfunction is presumed to be irreversible at the time of diagnosis because of the predominance of chronic inflammation. Therefore, the introduction of intense immunosuppressive therapy, as recommended for rapidly progressive MPA, may require careful consideration of the risk-benefit balance with the risk of infectious death.

MPO-ANCA and PR3-ANCA levels were measured at our institution to screen for ANCA-associated nephritis in cases of unexplained decline in renal function, regardless of the rate of renal function progression. When encountering a patient with unexplained renal dysfunction, ANCA, especially MPO-ANCA, should be measured, regardless of the rate of renal function decline. Slowly progressive MPA may also be latent in patients diagnosed with chronic nephritis syndromes, such as hypertensive nephrosclerosis or diabetic nephropathy, in cases in which renal biopsy has not been performed or ANCA has not been tested. No reports have examined the prevalence of subclinical MPA among all patients with chronic kidney disease and those undergoing dialysis, and such studies need to be conducted.

Limitation

During the course of Case 1, the patient did not develop RPGN. However, despite normal renal function 10 years ago, the subsequent progression of renal function is unknown. Given that the serum creatinine level had risen to 2.02 mg/dL at the initial consultation in 2022, the presence of RPGN cannot be completely ruled out. It cannot be denied that RPGN might have occurred prior to the 2022 blood test, followed by a slow decline in renal function. This hypothesis suggests that rapidly progressive MPA could transition to slowly progressive MPA without an apparent cause. To our knowledge, there have been no reports of RPGN in MPA leading to spontaneous remission or a similar state. However, it cannot be ruled out that the activity of MPA decreased following the removal of factors such as infections, drugs, tumors, or stress.

For both Case 1 and Case 2, we opted for steroid monotherapy instead of standard treatments including rituximab or cyclophosphamide. Therefore, caution may be necessary when generalizing the responsiveness of treatment in slowly progressive MPA from these cases.

## Conclusions

We encountered a case of slowly progressive MPA marked by a very gradual decrease in renal function. By comparing its clinical characteristics with those from a case of rapidly progressive MPA, which exhibited sclerotic pathology in the same period, the differences in disease activity became apparent. Slowly progressive MPA typically manifests lower disease activity that leads to chronic inflammation and a consequent slower decline in renal function. Unfortunately, slowly progressive MPA is often diagnosed too late. Thus, when evaluating a patient with unexplained renal dysfunction, irrespective of the decline rate, measuring MPO-ANCA to screen for slowly progressive MPA can be crucial.
